# The application value of lactate dehydrogenase in gynecological malignant tumors

**DOI:** 10.3389/fonc.2025.1731187

**Published:** 2026-01-05

**Authors:** Yanzhen Huang, Wenhao Zhang, Jue Hao, Ruizhen Zhu

**Affiliations:** 1 The First School of Clinical Medicine, Guangdong Medical University, Zhanjiang, Guangdong, China; 2 The Affiliated Yangjiang Hospital, Guangdong Medical University (People’s Hospital of Yangjiang), Yangjiang, Guangdong, China

**Keywords:** glycolysis, gynecologic malignancies, lactate dehydrogenase, LDHA inhibitors, tumor microenvironment

## Abstract

Lactate dehydrogenase (LDH) is the key enzyme catalyzing the interconversion between pyruvate and lactate, playing an indispensable role in cellular glycolysis. Five isoenzymes of this enzyme exist in the human body, each exhibiting distinct tissue distribution patterns and biological functions. Recent studies indicate that LDH isoenzyme LDHA exhibits significantly elevated expression in gynecological malignancies. Within the tumor microenvironment, cancer cells frequently endure hypoxic conditions. To counter this stress and satisfy the heightened energy requirements associated with rapid proliferation and invasion, cancer cells have to undergo a comprehensive reprogramming of their metabolic pathways, with notable changes observed in glucose metabolism. Multiple studies confirm that LDH expression levels are clearly associated with tumor proliferation, invasive metastasis capacity, clinical prognosis and chemotherapy sensitivity. Targeting LDHA inhibition effectively suppresses cancer cell growth. However, this strategy has yet to achieve clinical translation. The paper aims to systematically summarize the expression characteristics, molecular regulatory mechanisms, and clinical correlations of LDH in common gynecological malignancies. And explores the potential value of LDH as a novel diagnostic biomarker and therapeutic target.

## 1 Introduction

Gynecological malignancies rank among the leading causes of female mortality worldwide, with ovarian, endometrial, and cervical cancers being the most prevalent (1). With continuous advancements in diagnostic and treatment methods, the detection rates of endometrial cancer and cervical cancer in the early stages have improved, but ovarian cancer is usually diagnosed at an advanced stage, presenting significant treatment challenges with limited intervention options (2–4). Therefore, identifying efficient diagnostic biomarkers and therapeutic targets is crucial for enhancing patient survival and improving clinical outcomes. In recent years, tumor metabolic reprogramming has emerged as a frontier in tumor biology research. Tumor cells preferentially utilize glycolysis for energy production even in aerobic conditions, a metabolic characteristic termed the Warburg effect (5). As an indispensable enzyme governing glycolysis, LDH is integral to the execution of this metabolic adaptation mechanism. LDH is widely distributed in various human tissues and exists in five distinct isoenzyme forms (6). The LDH isoenzyme LDHA exhibits high expression in multiple tumors. It not only drives the transformation of pyruvate to lactate, promoting rapid ATP production, but also participates in maintaining the acidic characteristics of the tumor microenvironment, which synergistically promotes malignant behaviors such as tumor proliferation, invasion, and immune evasion (7). LDH levels demonstrate significant diagnostic and prognostic value across multiple gynecological malignancies and are closely associated with chemotherapy resistance (8). Experimental studies indicate that inhibiting LDHA effectively suppresses tumor cell growth capacity (9). A systematic summary of the role of LDH in gynecologic malignancies is currently lacking. This review elucidates its biological characteristics and potential pathogenic mechanisms, exploring its clinical applicability, and providing insights for diagnosis, prognosis, and treatment of these cancers.

## 2 Biological characteristics and functions of LDH

### 2.1 Molecular composition and tissue distribution of LDH

LDH is a redox enzyme present in multiple tissues, participating in the ultimate stage of glycolysis by catalyzing the reversible conversion between pyruvate and lactate. LDH primarily consists of M and H subunits. These two subunits assemble into five distinct tetrameric isoforms: two homotetramers, LDHB (H4) and LDHA (M4), along with three heterotetramers, namely LDH-2 (H3M), LDH-3 (H2M2), and LDH-4 (HM3) (6). The expression patterns of LDH isoenzymes display considerable diversity among different tissues. LDHB and LDH-2 are the predominant forms found in cardiac muscle and erythrocytes. The LDH-3 demonstrates elevated expression in cerebral tissue. While LDHA and LDH-4 are primarily located within liver and skeletal muscle (10). Recent research has identified a novel protein, designated LDHAα, which is 3 kDa larger than LDHA and expresses in various cancer cells. This protein enhances glycolytic activity, significantly boosting glucose uptake, lactate production, and tumor growth capacity (11). Additionally, researchers have identified a specific isoenzyme in testicular tissue named as LDH-C4. Early studies have classified it as a cancer testis antigen (12).

### 2.2 Functional characteristics of LDH

The primary function of LDH is to catalyze the reversible redox reaction between lactate and pyruvate. Under sufficient oxygen supply, LDHB predominantly catalyzes the oxidation of lactate to pyruvate which is channeled into the mitochondrial TCA cycle, followed by the oxidative phosphorylation process, enabling efficient ATP production. In hypoxic conditions, pyruvate cannot enter the TCA cycle. Instead, it is reduced to lactate by LDHA, simultaneously oxidizing NADH to NAD^+^. This process ensures glycolysis can continue under oxygen-deprived conditions (6). Notably, even under normal oxygen partial pressure, tumor cells preferentially oxidize glucose to lactate via aerobic glycolysis to generate rapid, low-yield ATP rather than entering mitochondrial oxidative phosphorylation—a phenomenon termed the Warburg effect (5). Extensive research indicates that LDHA expression is mostly upregulated in cancer cells, potentially linked to the metabolic reprogramming of tumor cells (13). Related studies in T cells further reveal that the dynamic equilibrium between LDHA and LDHB is pivotal for governing normal cellular activities. Specific knockout of LDHA inhibits glycolytic flux, cell proliferation, and differentiation processes. Conversely, while knockout of LDHB partially boosts glycolysis but also impedes T cell differentiation. An appropriate ratio of LDHA to LDHB is crucial for maintaining glycolytic activity, redox homeostasis, and normal cellular physiological functions (14).

## 3 LDH expression in gynecological malignancies

In ovarian cancer, transcription levels of critical enzymes in glycolysis particularly LDHA and pyruvate kinase M show significant positive correlations with RAD23A. While elevated RAD23A enhances ovarian cancer cell resistance to niraparib (15). In addition, LDHB promotes PD-L1 expression by regulating H3K18la in the PD-L1 promoter region, thereby aiding ovarian cancer cells in immune evasion (16).

LDHA exhibits overexpression in both tumor tissues and cell lines in endometrial carcinoma, serving as a potential biomarker for accurately distinguishing normal endometrium from carcinoma (17). LDHA expression intensity correlates closely with tumor histological grade and myometrial invasion depth. Patients with higher grades typically exhibit elevated LDHA levels. Downregulating LDHA expression promotes apoptosis and inhibits EC cell proliferation and invasiveness (17). Research indicates that STAT3 directly binds to the LDHB promoter region, enhancing tumorigenic properties in endometrial carcinoma cells by upregulating malate MDH2 expression (18).

Research by Fengxia Zha et al. indicates that the LDH-C4 subtype is highly expressed in exosomes from cervical cancer patients. Its expression demonstrates significant associations with tumor grade, disease stage, lymph node metastasis, and overall survival, serving as an independent factor predicting poor prognosis in cervical cancer (19). Although most studies indicate that LDHA promotes carcinogenesis by enhancing glycolysis, and its inhibition typically suppresses tumor growth, the opposite trend is observed in cervical cancer. Chaoran Jia et al. discovered that inhibiting LDHA under conventional culture conditions markedly reduced aerobic glycolysis, cell survival, and proliferation in cervical cancer cells. But the accumulated cytoplasmic pyruvate could be shunted into mitochondria to generate oxaloacetate. This process activated AMPK, maintaining mitochondrial homeostasis and enhancing resistance to reactive oxygen species-induced ferroptosis. Ultimately enhancing cervical cancer cell survival, suggesting that targeting LDHA inhibition may not be an optimal therapeutic strategy for cervical cancer (20).

Elevated LDH levels are also observed in certain rare gynecological malignancies. For instance, Ziren Fen et al. reported a rare case of ovarian germ cell dysplasia where common tumor markers like serum CA125, AFP, and CEA remained within normal ranges, yet LDH was significantly elevated at 1112 U/L, suggesting LDH may hold special diagnostic value in such cases (21). PLFGT is a clinically uncommon malignant tumor with low incidence. A retrospective analysis of 13 PLFGT patients revealed elevated serum LDH in 10 cases, while only 2 exhibited increased CA125 levels. This suggests that preoperative serum LDH testing may serve as a potential tumor marker for PLFGT (22). It is noteworthy that the clinical utility of LDH in rare gynecologic malignancies relies mainly on small-sample retrospective analyses, and its reliability as a diagnostic biomarker requires further validation. However, due to the rarity of these tumors and limited clinical data, elevated LDH levels remain valuable in their diagnosis and treatment evaluation.

## 4 Potential mechanisms of LDH involvement in gynecological malignancy progression

### 4.1 Glucose metabolic reprogramming and enhanced glycolysis

Unlike normal cells, tumor cells tend to take up large amounts of glucose and convert it into lactate even in oxygen-rich environments, while simultaneously reducing oxidative phosphorylation levels. Otto Warburg initially attributed this phenomenon to mitochondrial dysfunction (23). Subsequent research has progressively refined this understanding. Hypoxia emerges as a fundamental characteristic of the tumor microenvironment due to local blood supply insufficiency caused by rapid tumor growth (24). Furthermore, tumor cells exhibit biological traits such as unlimited proliferative potential, persistent angiogenesis, anti-apoptotic capacity, insensitivity to inhibitory signals, and invasive metastasis. To acquire the energy and nutrients required for survival and maintenance of these essential functions, tumor cells must undergo metabolic reprogramming (25). Tumor metabolic reprogramming encompasses multiple aspects including glucose utilization, glutamine breakdown, lipid uptake and oxidation, and branched-chain amino acid metabolism, with alterations in glucose metabolism being most prominent. This is because glycolysis produces essential biosynthetic substrates and intermediates required for the production of amino acid, nucleotide, and lipid synthesis beyond of providing energy (26). Although complete oxidative phosphorylation of glucose yields approximately 36 ATP molecules per glucose molecule, while glycolysis generates only 2 ATP molecules, lactate production occurs roughly two orders of magnitude faster than oxidative phosphorylation. This facilitates rapid fulfillment of the energy demands for tumor cell proliferation (27), providing a rational physiological explanation for tumors’ preference for aerobic glycolysis.

Glucose metabolic reprogramming is regulated by multifaceted mechanisms, including critical metabolic enzyme activities, transcriptional regulator actions, the triggering of signaling cascades and the modulation of metabolic feedback. Within the group of glycolysis-related enzymes, HK2 and LDHA play particularly prominent roles. The binding of HIF-1α to a Hypoxia-Response HRE present in the HK2 promoter leads to the subsequent activation of this gene’s transcription. This allows tumor cells to sustain an elevated glycolytic flux despite the presence of adequate oxygen (28). As a core regulator of tumor invasion and energy metabolism adaptation, HIF-1α directly binds to HREs of glycolysis-related genes (GLUT1, HK2, PDK1, LDHA, etc.), increasing expression of key glycolytic enzymes and suppressing mitochondrial aerobic metabolism, playing a pivotal role in cancer cell growth and metastasis (29). Moreover, HIF-1α serves as a central node integrating multiple transcriptional regulation and signaling pathways (30). Ma et al. demonstrated that CREB1 activates the HIF-1 signaling axis by upregulating WNK1, thereby promoting proliferation and metastatic behavior in ovarian cancer cells (31). Furthermore, acting as a key regulator, Myc drives the expression of numerous glycolysis-related genes through direct transcriptional upregulation, including LDHA, significantly enhancing tumor cell glycolytic capacity (32).

### 4.2 Regulation of the tumor microenvironment

LDH not only directly promotes tumor cell proliferation by catalyzing glycolytic reprogramming, but its enzymatic activity and downstream glycolytic metabolites also play a pivotal role in shaping and maintaining the tumor microenvironment (TME). TME, a complex cellular ecosystem, chiefly comprises malignant tumor cells alongside diverse non-malignant cells including fibroblasts, macrophages, various immune cells, mesenchymal stem cells, and endothelial cells. These cells collectively sustain tumor growth and proliferation through metabolic crosstalk (33). Verma et al. demonstrated that LDHA expression is significantly higher in cancer cells than in non-cancerous cells. LDH inhibitors can rebalance glycolytic competition between tumor cells and infiltrating regulatory Tregs, disrupting Tregs functional stability (34). Within tumor tissues and their microenvironments, Tregs suppress the activity of other immune cells, aiding tumor immune evasion and promoting growth (35).

The substantial lactate produced during glycolysis further promotes the progression of gynecological malignancies by modulating the tumor microenvironment (36). Microenvironment acidification directly impairs the function of CD8^+^ and CD4^+^ T lymphocytes, and suppresses T cell proliferation and weakens the cytotoxicity of NK cells, consequently promoting the evasion of immune surveillance by malignant cells (37). Tumor-associated macrophages (TAMs), abundant in the TME, serve as key mediators of immunosuppression. Xiao die Li et al. found that in endometrial cancer, lactate drives M2 polarization of TAMs, which enhances tumor aggressiveness by facilitating EMT and angiogenesis (38). Lactic acid generated via LDH-mediated pathways also modulates interactions between tumor cells and vascular endothelial cells (36). Studies indicate that lactic acid enhances HIF1α stability in tumor-associated endothelial cells, subsequently inducing the expression of VEGF, which facilitates angiogenesis in ovarian cancer (39, 40). Cancer-associated fibroblasts (CAFs), one of the mainly matrix cell types in the TME, extensively communicate with cancer cells through signaling molecule secretion or intercellular adhesion. They also remodel the extracellular matrix and modulate immune cells, collectively promoting tumor proliferation, metastasis, and immune evasion (41). Additionally, PARP-1 activation by reducing the NAD^+^/NADH ratio and downregulates p62 expression which acts as an inhibitor of CAF activation and tumor progression (42). Conversely, overexpression of GLUT1 in CAFs enhances glucose transport and accelerates glycolysis and elevates lactate output, promoting ovarian cancer cell proliferation and migration via the TGF-β1/p38/MMP2/MMP9 signaling pathway (43). Accumulated lactate in the TME also enhances tumor cell resistance to ferroptosis, making a contribution to tumor cell proliferation, metastasis, and drug resistance (44).

Lactate is not merely a metabolic byproduct, but also serves as a precursor for epigenetic modifications. Lactylation, a novel post-translational modification where lactate binds lysine residues, drives tumor progression by accelerating proliferation, dissemination and chemoresistance (37). A study in investigating ovarian cancer resistance to niraparib revealed that H4K12 lactylation upregulates RAD23A via super-enhancers, potentiating DNA repair and fostering chemoresistance (15). Another study indicated that L-lactic acid increases lactylation modification of the DCBLD1 protein, directly stabilizing DCBLD1 and enhancing its transcription and expression, ultimately boosting cervical cancer cell growth, invasion, and treatment resistance (45).

## 5 Clinical application value of LDH in gynecological malignancies

### 5.1 Diagnostic and prognostic assessment value

The elevated expression of LDH in tumor tissue provides valuable information for cancer diagnosis. In its initial phases, ovarian carcinoma frequently demonstrates a lack of characteristic clinical manifestations (46). CA125 and HE4 are commonly used in clinical practice for ovarian cancer diagnosis. While serum CA125 is elevated in most patients, its sensitivity for early-stage disease is low (47). HE4, as a more specific biomarker, is often combined with CA125 to improve accuracy (48). Unlike these conventional markers that indicate tumor origin or monitor recurrence, LDH reflects metabolic reprogramming and immunosuppressive microenvironment features, collectively representing tumor metabolic activity and invasiveness, thereby complementing traditional biomarkers. Studies indicate that combining ovarian malignant tumor risk prediction models with LDH testing aids in distinguishing early-stage ovarian cancer from benign ovarian lesions, combining LDH with traditional biomarkers may enhance early ovarian cancer detection (49). However, existing evidence mainly derives from retrospective studies in diagnosed patients, likely missing undetected early cases due to diagnostic challenges. Thus, LDH remains a preliminary screening candidate, lacking prospective validation in asymptomatic populations. Research on LDH is more established in ovarian than in cervical or endometrial cancers. Although LDHA shows diagnostic potential for endometrial carcinoma (17), current *in vitro* evidence is inadequate to support its clinical reliability as a novel biomarker.

As an indicator of tumor metabolic activity and burden, LDH demonstrates prognostic potential in ovarian cancer. A study revealed that serum LDH concentrations in ovarian cancer patients are significantly higher than in those with benign tumors, and these levels correlate closely with tumor clinical staging and pathological grading (50). Numerous studies have confirmed that elevated LDH levels are closely associated with adverse outcomes in cervical cancer. A study involving 908 cervical cancer cases demonstrated that serum LDH was universally elevated, with higher LDHA expression typically associated with poorer prognosis (51). A multivariate logistic regression analysis by Qiuyuan Huang et al. revealed a significant positive correlation between LDH levels and lymphatic metastasis in cervical cancer cohort. Elevated LDH was associated with increased likelihood of deep stromal invasion and lymphovascular invasion, suggesting its potential as a tumor marker and treatment monitoring indicator (52). Further research indicates that regardless of FIGO stage in cervical cancer, overexpression of both LDHA and PFKP is significantly correlated with poorer overall survival, elevated likelihood of recurrence, and higher mortality risk, suggesting their importance in assessing disease progression and prognosis (53). In light of this, clinicians can accurately assess disease progression by integrating LDH levels with gynecological malignancy-specific markers and imaging data.

### 5.2 Guiding individualized treatment

Treatment modalities for gynecologic malignancies principally include surgery, systemic chemotherapy, radiotherapy, and immunotherapy (54). For advanced or recurrent cases post-surgery, chemotherapy and immunotherapy often serve as core strategies. However, low drug sensitivity remains a major challenge (55). The development of drug resistance is a key factor in treatment failure and disease recurrence (56), making the need for personalized treatment strategies increasingly urgent. Multiple studies suggest associations between serum LDH levels and drug efficacy and resistance. As a case in point, Asami Ikeda et al. reported that elevated serum LDH and NLR may predict platinum resistance and poor prognosis in ovarian cancer patients, providing reference for personalized treatment (57). The KELIM score serves as a reliable indicator for assessing chemotherapy sensitivity in ovarian cancer, with higher scores suggesting better treatment response (58). Among patients with poor KELIM scores, those with elevated LDH levels derive minimal benefit from first-line bevacizumab therapy. Lower LDH levels (≤ 210 U/L) serve as an independent predictor of therapeutic benefit from this agent in advanced ovarian cancer (59). Thus, dynamic monitoring of LDH levels before or during treatment may provide crucial guidance for optimizing chemotherapy regimens.

### 5.3 Potential for targeted intervention therapy

As a key enzyme regulating glycolysis in tumor cells, LDHA is emerging as a promising and prospective focus in developing novel anticancer therapeutics overcoming drug resistance (9). Studies indicate that isoprolactone reduces glucose uptake and lactate production in cisplatin-resistant ovarian cancer cells by targeting LDHA inhibition, increasing the susceptibility of cisplatin, suppressing tumor growth and overcoming resistance (60). Research by Y Tian et al. indicates that miR-1271 suppresses proliferation and metastatic capacity in endometrial cancer cells by downregulating LDHA expression, providing theoretical support for developing novel biomarkers and therapeutic strategies (61). In cervical cancer, inhibiting LDHA induces G2/M phase cell cycle arrest and activates the mitochondrial-mediated apoptosis pathway (62). On the contrary, Chaoran Jia et al. demonstrated that LDHA deficiency paradoxically suppresses glucose-starvation-induced ferroptosis, consequently stimulating cell multiplication and the process of oncogenesis (20). Accordingly, whether LDHA inhibition strategies are applicable for targeted therapy in cervical cancer warrants further investigation.

## 6 Current research limitations and future challenges

Current research on LDH faces certain limitations and challenges. LDH is not only highly expressed in various tumor cells but also widely distributed in non-tumor cells within tumor tissues and multiple normal tissues. consequently, its levels can be elevated under various physiological and pathological conditions (63, 64). This extensive tissue distribution pattern limits its utility as a specific biomarker for gynecological malignancies. For this reason, developing detection techniques targeting LDH isoenzymes holds promise for enhancing sensitivity and specificity in diagnosing gynecological cancers. Diverse LDH activity assays have been established, including colorimetric, spectrophotometric, electrochemical, and fluorometric methods (65). While these methods generally meet routine testing requirements, significant inconsistencies exist among results obtained using different approaches. Multiple factors during detection, including substrate concentration, sample coloration, and other impurities, may interfere with LDH assay outcomes (65). The main of current challenge is improving LDH detection accuracy, enabling real-time dynamic monitoring, and establishing highly sensitive and specific LDH isoenzyme analysis systems.

Although inhibiting LDHA can delay tumor progression, the development of LDHA inhibitors remains in the exploratory phase and has not yet achieved clinical translation. Because inhibiting its activity may simultaneously disrupt the energy metabolism homeostasis of normal cells, potentially triggering significant adverse reactions (66). Besides, even when the glycolytic pathway in tumor cells is inhibited, they can activate alternative metabolic pathways to sustain energy supply and promote continued tumor progression (26). For instance, tumor cells can upregulate LDHB to reverse the reaction catalyzed by LDHA, thus regenerating pyruvate and NADH from lactate and NAD^+^ to fuel their energetic and anabolic needs for mitosis (67). Beyond sustaining cancer cell growth through glycolysis, LDHA orchestrates tumor metastasis and immune evasion by modulating multiple signaling pathways. And its product, lactate, also promotes tumor proliferation and induces immunosuppression (36). Therefore, targeting LDH alone may prove insufficient to completely block tumor progression. A critical consideration is that LDHA inhibition has been found to promote the survival and proliferation of cervical cancer cells under energy stress conditions (20), which limits the broad applicability of LDHA-targeted therapy across gynecological malignancies. This observation highlights the significant impact of energy stress within the tumor microenvironment on therapeutic response. Although similar effects have not yet been reported in ovarian or endometrial cancers, the metabolic plasticity and adaptive capacity of tumor cells may enable them to develop robust escape mechanisms. Therefore, the extension of LDHA inhibition as a treatment strategy for gynecological cancers warrants caution and further investigation. A promising future avenue is to pursue integrated strategies that combine metabolic modulation with tumor microenvironment intervention (68).

## 7 Conclusion

LDH shows promise in the diagnosis, prognostic assessment, and guidance of individualized treatment for gynecological malignancies. However, current evidence is largely focused on ovarian and cervical cancers, with limited studies in endometrial carcinoma, warranting further investigation into its clinical role. As a key rate-limiting enzyme in the aerobic glycolysis of tumor cells, the inhibition of LDHA activity has emerged as a novel target in cancer therapy. Currently LDHA inhibitors have entered the preclinical research stage. Nevertheless, LDHA inhibition may promote tumor growth in cervical cancer models, suggesting limited applicability across gynecological tumors. Future research should clarify the metabolic network in the tumor microenvironment and use biomarkers to monitor energy status, enabling more precise and combined treatments.
